# The Potential of Clover Green Amendment, Associated with Biochar, Activated Carbon or Ochre, for the Phytoremediation, Using *Populus x. canescens*, of a Former Mine Technosol

**DOI:** 10.3390/plants10071374

**Published:** 2021-07-05

**Authors:** Manhattan Lebrun, Sylvain Bourgerie, Domenico Morabito

**Affiliations:** INRA USC1328, LBLGC EA1207, Faculty of Sciences, University of Orléans, 45067 Orléans, France; sylvain.bourgerie@univ-orleans.fr (S.B.); domenico.morabito@univ-orleans.fr (D.M.)

**Keywords:** (in)organic amendments, metal(loid)s, phytostabilization, poplar

## Abstract

Metal(loid) soil pollution resulting from past and present mine activities is a serious environmental and health issues worldwide. Therefore, the remediation of those polluted areas has been a growing research interest over the last decades, especially the assisted phytoremediation. In this study, a pot experiment was set up, using a former mine technosol, highly polluted by As and Pb, to which biochar, activated carbon, or ochre was applied, alone or in combination to clover green amendment. Following amendment application, *Populus x. canescens* cuttings were planted. Results showed that all four amendments reduced soil acidity. However only the first three amendments immobilized As and Pb, while the green amendment drastically mobilized those two pollutants and none of the amendments improved plant growth. In conclusion, the association of clover green amendment to biochar, activated carbon, or ochre did not appear as an efficient remediation strategy in this case; although the aging of the amendments and degradation of the green amendment in the soil with time could have positive outcomes.

## 1. Introduction

Soil is at the center of our life. It provides food and other ecosystem services and thus a good soil condition is essential. However, due to anthropogenic activities, such as mining extractions, industrial activities, fertilizer, and pesticide uses in agriculture…, soil states are degraded. Notably, many soils across the world are polluted by metals and metalloids [1]. Soil pollution by metal(loid)s pose a serious threat to the environment and human health. Indeed, polluted soils often lack vegetation and metal(loid)s are easily dispersible, thus contamination can spread to the surrounding environment through wind erosion, and towards underground water through leaching [2]. Contaminated soils originating from mine extraction activities are usually characterized by huge amounts of sterile soils, having no structures, which make them highly subject to collapse. Moreover, metal(loid)s are highly toxic and carcinogenic, notably As, Pb, Cd, and Hg have been registered as the four most toxic metal(loid)s [3]. Consequently, there is a need to remediate such former mine extraction sites highly polluted. For decades, physical and chemical remediation techniques have been implemented due to their fast results. However, such techniques are expensive, difficult to install on a large scale, and are disruptive to soils [1,4]. For this reason, phytoremediation, a biological method for soil remediation, is now more and more studied and used. This technique uses plants, and associated microorganisms, to reduce the toxicity and negative effects of the pollutants [5]. Phytoremediation is divided in two main processes: phytoextraction and phytostabilization. In the phytoextraction, plants are used to extract pollutants from the soil. They take them up through their roots and translocate them in their upper parts. The aerial biomass is harvested, which removes the contamination from the site. However, it requires the proper disposal of the contaminated biomass, adding an additional cost to the process [1,6]. On the contrary, the phytostabilization technique does not remove pollution but stabilizes it. In this context, pollutants are immobilized at the root zone—i.e., the rhizosphere—through absorption inside the roots, adsorption on the root surface, or through root exudate complexation. Additionally, plants will stabilize the soil limiting erosion [6,7]. The success of the remediation process will depend on the ability of the plant to develop thus the selection of a tolerant and fast growing plant species is essential. Among different plants that can be used, studies showed that poplars were efficient phytostabilization plants. For instance, Nissim et al. [8] performed a field experiment with four species—one poplar, willow, hemp, and alfalfa—on an abandoned area contaminated with Cu, Pb, and Zn. They concluded that under the Mediterranean climate conditions tested poplar and willow were efficient in the phytoremediation. Similarly, Redovnikovic et al. [9] demonstrated that *Populus nigra* could be used for the phytoextraction of Cd and the phytostabilization of Pb. Another advantage of poplars is their high biomass production, which could be used for bioenergy production, adding an economic profit [5]. In this study, one poplar species was tested, *Populus x. canescens*, which has a deep plunging root system, and thus helps to prevent soil from collapsing.

However, for plants to establish, the soil needs to possess nutrients and proper growing conditions, which are often not met in the case of metal(loid) contaminated soils, presenting extreme pH, low nutrient, and organic matter contents and high metal(loid) concentrations. That is why amendments must be applied, which is considered as one of the most promising field remediation techniques [4]. In their study, Wang et al. [6] stated that applying amendments had five direct effects on soil properties: (i) increase soil organic matter content, (ii) increase soil nutrient content and availability, (iii) rise soil cation exchange capacity, (iv) improve soil water holding capacity, and (v) reduce metal(loid) availability and leaching. This indirectly ameliorates plant growth. Many amendments can be used, organic and inorganic, but four gathered attention over the last years and will be the subject of this study: biochar, activated carbon, red mud and green amendment.

Biochar is one of the three products, together with oil and gas, of the pyrolysis of biomass. It is a porous material, characterized by high carbon content, generally above 60%, an elevated surface area, an alkaline pH, a high cation exchange capacity and the presence of negatively charged functional groups on its surface [4,10,11]. Due to such properties, biochar has been shown to improve soil conditions, i.e., reduce soil acidity, increase nutrient and organic matter contents, as well as to immobilize metal(loid)s [12,13,14]. Activated carbon is also a porous and carbon-rich material, derived from a pyrolysis process. However, it has been subjected to an additional treatment compared to biochar, i.e., an activation. The activation can be physical or chemical and performed after the pyrolysis or prior to it, on the raw feedstock [15,16]. Such activation is made in order to ameliorate properties of the material and thus its effects on the soil and pollutants. The beneficial effects of activated carbon were shown in previous studies [17,18,19]. Contrary to biochar and activated carbon, red mud is an inorganic amendment, considered as waste generated from alumina production [10]. It is characterized by an alkaline pH and a high surface area [20,21]. As a red mud-like product, ochre is obtained from charcoal mine and was shown beneficial for both metals and metalloids, as well as for soil properties [22,23]. Finally, a high amount of crop waste is generated in the agriculture; such vegetal biomass contains nutrients, which could be beneficial to plants. Therefore, green manure in the form of crushed vegetal biomass has been studied and showed positive effects on organic matter content, nutrient levels, metal(loid) immobilization, and plant growth [24,25,26].

The amendments biochar, activated carbon, and red mud are mainly a source of alkalinity and carbon, and are used for metal(loid) immobilization; however, they are not a source of nutrients per se. Thus, they can be added together with the green amendment, a high nutrient source.

In this context, the objectives of this study were to evaluate the effects of clover straw, as a green amendment, associated to biochar, activated carbon or ochre, on (i) soil physicochemical properties; (ii) As and Pb stabilization; and (iii) *Populus x. canescens* growth and metal(loid) accumulation.

## 2. Results

A mesocosm experiment was performed using a former mine technosol (PG) highly contaminated with As and Pb, to which different additives were applied, i.e., biochar (Bc), ochre (Oc), activated carbon (AC), and clover green amendment (GA). On these different substrate *Populus x. canescens* were grown for 45 days. 

### 2.1. Soil Pore Water Physicochemical Properties

At the beginning of the experiment (T0), the SPW of PG was acidic, in both –GA and +GA conditions (Table 1). In the treatments without GA (i.e., –GA), pH increased with biochar (+1.8 units) and ochre (+1.6 units). In the treatments with GA (i.e., +GA), all three amendments increased SPW pH. Biochar (+2.3 units) and ochre (+2 units) induced a higher increase than activated carbon (+1.2 units). When comparing conditions −GA and +GA, a green amendment effect was observed for PG+Oc and PG+AC, in which GA induced a rise in SPW pH, by 0.3 units and 0.9 units, respectively ( Table 1 and Table S1).

At the end of the experiment (T45), PG was still acidic. In the treatments without the green amendment, SPW pH increased with all the amendments and a higher increase was measured with biochar (+1.4 units) and ochre (+1.2 units) than with activated carbon (+1.1 units). In the +GA conditions, there was no effect of biochar, ochre, and activated carbon. Moreover, there was no significant difference between the treatments −GA and +GA, demonstrating no green amendment effect on SPW pH after 45 days (Table 1 and Table S1).

Finally, in three cases, SPW pH increased between T0 and T45, i.e., PG+GA, PG+AC-GA, and PG+AC+GA, while in one treatment, PG+Bc+GA, it decreased.

The SPWs were also analyzed for electrical conductivity (EC). At T0, SPW EC in PG was 2072 µS.cm^−1^ without GA. SPW EC increased 1.3-fold and 1.5-fold with biochar and ochre, respectively (Table 1). In the treatments with GA, biochar and ochre induced a 1.5-fold increase in SPW EC. Moreover, adding the green amendment increased SPW EC in all conditions, by 1.3-fold in PG, 1.4-fold in PG+Bc, 1.3-fold in PG+Oc, and 1.3-fold in PG+AC (Table 1 and Table S1).

At T45, in the treatments without GA, SPW EC of PG 3961 µS.cm^−1^ and only the application of activated carbon induced a 41% decrease in SPW EC (Table 1). In the treatments with GA, there was no effect of biochar, ochre and activated carbon. Moreover, at T45, green amendment had no effect (Table 1 and Table S1).

Finally, SPW EC increased between T0 and T45 in PG, PG+Oc-GA, and PG+AC+GA.

The redox potential (Eh) in the SPW of PG, without GA, was 364 mV, and it only decreased with ochre, by 21% (Table 1). In the condition with GA, SPW Eh of PG was 3030 mV and decreased with biochar by 14%, and ochre by 20%. At that time, applying green amendment decreased Eh in PG+Bc (by 15%) and PG+AC (by 35%) (Table 1 and Table S1).

At T45, in the –GA conditions, SPW PG had an Eh of 387 mV and all the amendments decreased Eh: biochar by 24%, ochre by 24%, and activated carbon by 23%. No difference between the three amendments was found (Table 1). In the conditions with the green amendment, SPW Eh of PG was 268 mV and no effect of biochar, ochre and activated carbon was measured; but PG+Oc had a lower Eh compared to PG+Bc. At that time, the addition of green amendment decreased SPW Eh by 38% in PG+Bc and 10% in PG+AC (Table 1 and Table S1).

Finally, in the condition PG+AC-GA, SPW Eh was observed to decrease between T0 and T45 (Table 1 and Table S1).

### 2.2. Soil Pore Water Metal(loid) Concentrations

The SPWs were analyzed for As and Pb concentrations, the most important contaminants of the site, and Fe concentrations, which can be added by the amendments.

At T0, in the −GA conditions, SPW As concentration was 0.19 mg.L^−1^ in PG and all the amendments decreased SPW As concentrations, biochar by 63%, ochre 74%, and activated carbon 95% (Table 2). In the conditions with GA, SPW As concentration of PG was 0.37 mg.L^−1^ and only ochre decreased it, by 86%. Adding the green amendment had a negative effect on As mobility: GA induced an increase in SPW As concentration in PG (2-fold), PG+Bc (5-fold), and PG+AC (46-fold) (Table 2 and Table S1).

At T45, in the conditions without GA, in the SPW of PG, As concentration was 0.30 mg.L^−1^ while in the +GA condition, it was 0.58 mg.L^−1^ (Table 2). In both cases, there was no effect of biochar, ochre and activated carbon. At that time, SPW As concentration increased following the addition of GA on PG (2-fold), PG+Oc (4-fold), and PG+AC (3-fold) (Table 2 and Table S1).

Finally, SPW As concentration increased between T0 and T45 in PG+Oc-GA, PG+Oc+GA, and PG+AC-GA.

At the beginning of the experiment, SPW Fe concentration was 0.32 mg.L^−1^ in PG without GA (Table 2) and only biochar addition decreased it, by 50%. In treatments with GA, SPW PG had 2.53 mg.L^−1^ Fe and all amendments decreased SPW Fe concentrations, biochar by 87%, ochre by 92% and activated carbon by 85%, with no significant difference between the three amendments. The addition of GA increased SPW Fe concentration in PG and PG+AC, by 8-fold and 1.8-fold, respectively (Table 2 and Table S1).

At T45, when no green amendment was added, SPW Fe concentration in PG was 4.4 mg.L^−1^ and there was no effect of adding biochar, ochre, and activated carbon (Table 2). In the conditions +GA, SPW of PG had a Fe concentration of 28.58 mg.L^−1^ and only ochre increased it by 5-fold. At that time, adding green amendment increased Fe concentrations in the SPW of PG+Oc (621-fold) and PG+AC (56-fold) (Table 2 and Table S1).

Finally, SPW Fe concentrations increased between T0 and T45 in PG+Oc+GA and PG+AC+GA.

The SPW of PG treatment, without GA, had a Pb concentration of 4.4 mg.L^−1^ and adding biochar, ochre and activated carbon did not influence Pb concentration. However, in the +GA conditions, SPW Pb concentration was 11.8 mg.L^−1^ in PG and biochar, ochre and activated carbon decreased it; the addition of activated carbon led to a higher decrease (60%) compared to biochar (50%) and ochre (47%). At that time, green amendment addition increased Pb mobility in PG (2.7-fold) and PG+Oc (1.7-fold) (Table 2 and Table S1).

At the end of the experiment, in conditions without GA, SPW Pb concentration was 3.7 mg.L^−1^ in PG and similarly to T0, there was no effect of the biochar, ochre and activated carbon amendments (Table 2). In the conditions with GA, SPW Pb concentration in PG was 4.1 mg.L^−1^ and applying biochar and ochre decreased it, by 85% and 70%, respectively. At that time, GA application led to a decrease in SPW Pb concentration in PG+Bc (78%) and PG+Oc (68%) (Table 2 and Table S1).

Finally, a decrease in SPW Pb concentration between T0 and T45 was observed in PG+GA, PG+Bc-GA, PG+Bc+GA, PG+Oc+GA, and PG+AC-GA.

### 2.3. CaCl_2_ Metal(loid) Concentrations

In addition to the evaluation of metal(loid) mobility, through the measure of SPW concentration, metal(loid) availability was assessed through CaCl_2_ extractions performed on soils sampled at the end of the experiment.

In PG, with or without GA, As was not detected in CaCl_2_ extractions, while small concentrations were measured in PG+Bc, with and without GA, as well as PG+Oc+GA (Table 3). For CaCl_2_-As concentrations, biochar, ochre and activated carbon amendments had no effect. Similarly, green amendment did not affect As availability (Table 3 and Table S1).

In the –GA conditions, CaCl_2_-Fe concentration was 3.37 mg.kg^−1^ in PG and there was no effect of biochar, ochre and activated carbon (Table 3). In the conditions with GA, Fe available concentration in PG was 3.99 mg.kg^−1^ and only the activated carbon amendment decreased it, by 43%. Moreover, the application of the green amendment decreased Fe availability in PG+Bc and PG+AC, by 42% and 20%, respectively (Table 3 and Table S1).

Finally, among the three metal(loid)s, Pb had higher available concentrations than As and Fe. On PG, without GA, Pb available concentration was 114 mg.kg^−1^ and only ochre amendment induced a 40% decrease of Pb availability (Table 3). In the +GA conditions, CaCl_2_-Pb concentration was 90 mg.kg^−1^ in PG and the three amendments—biochar, activated carbon, and ochre—had no effect. Adding green amendment on PG+AC reduced Pb availability by 24% (Table 3 and Table S1).

### 2.4. Plant Leaf Pigments

**Figure 1:**
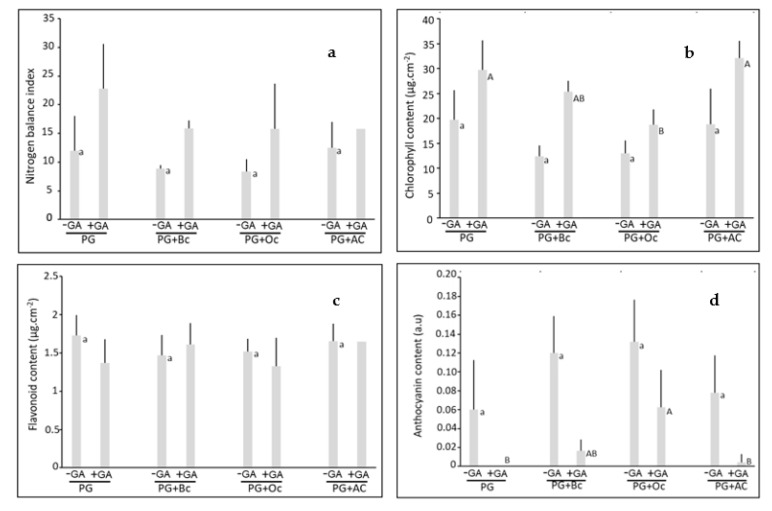
Nitrogen balance index (**a**) and pigment contents (chlorophyll (µg.cm^−2^) (**b**), flavonoid (µg.cm^−2^) (**c**) and anthocyanin (a.u) (**d**) determined on the leaves of *Populus ×. canescens* after 45 days of growth on Pontgibaud technosol alone (PG) or amended with biochar (PG+Bc), ochre (PG+Oc), and activated carbon (PG+AC), associated or not with green amendment (+/− GA). Small letters indicate a significant difference between the treatments without green amendment while capital letters indicate a significant difference between the conditions with the green amendment (*p* < 0.05) (n = 5 ± SE).

After 45 days of growth on the different substrates, leaf pigments were measured. The nitrogen balance index (NBI) was not affected by biochar, ochre and activated carbon, in −GA and +GA conditions (Figure 1a). The addition of GA increased NBI values on PG+Oc by 2-fold (Figure 1a, Table S1).

On PG, without GA, chlorophyll content was 20 µg.cm^−2^ and biochar, ochre and activated carbon had no effect (Figure 1b). In the +GA conditions, chlorophyll content of plants grown on PG was 30 µg.cm^−2^ and ochre amendment decreased it by 37%. The green amendment increased chlorophyll content in all treatments: PG (1.5-fold), PG+Bc (2.1-fold), PG+Oc (1.4-fold), and PG+AC (1.7-fold) (Figure 1b, Table S1).

The flavonoid content was not affected by any of the treatments, biochar, activated carbon, ochre, and green amendment (Figure 1c, Table S1).

Finally, in plants grown on PG, without GA, anthocyanin level was 0.06 a.u. and not affected by the application of biochar, ochre or activated carbon (Figure 1d). In the conditions containing GA, anthocyanin content was very low, except with ochre. Finally, the application of GA decreased anthocyanin content in all treatments, PG, PG+Bc (87%), PG+Oc (53%), and PG+AC (95%) (Figure 1d, Table S1).

### 2.5. Plant Stem Length, Dry Weight, and Metal(loid) Concentrations

**Figure 2:**
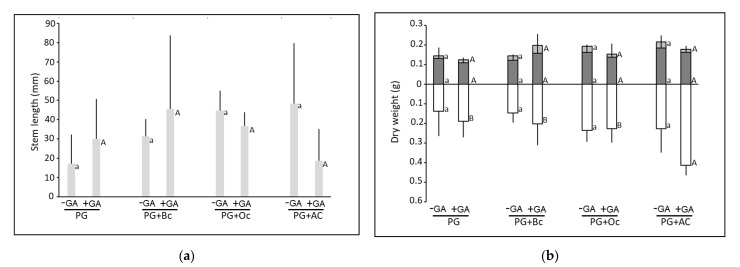
Stem length (mm) (**a**) and leaf (light grey), stem (dark grey) and root (white) dry weight (g), (**b**) of *Populus ×. canescens* after 45 days of growth on Pontgibaud technosol alone (PG) or amended with biochar (PG+Bc), ochre (PG+Oc) and activated carbon (PG+AC), associated or not with green amendment (+/− GA). Small letters indicate a significant difference between the treatments without green amendment while capital letters indicate a significant difference between the conditions with the green amendment (*p* < 0.05) (n = 5 ± SE).

The different treatments, biochar, ochre, activated carbon and green amendment, had no effect on stem length (Figure 2a).

Similarly, leaf and stem dry weight was not affected by biochar, ochre, activated carbon and green amendment (Figure 2b). However, root dry weight differed among treatments. In more details, on PG, without GA, root dry weight was 0.14 g and not affected by biochar, activated carbon and ochre. In +GA conditions, root dry weight of plants grown on PG was 0.19 g and ochre amendment increased it by 2-fold. Green amendment addition led to an improvement of root dry weight in PG+AC, by 1.8-fold (Figure 2b, Table S1).

**Figure 3:**
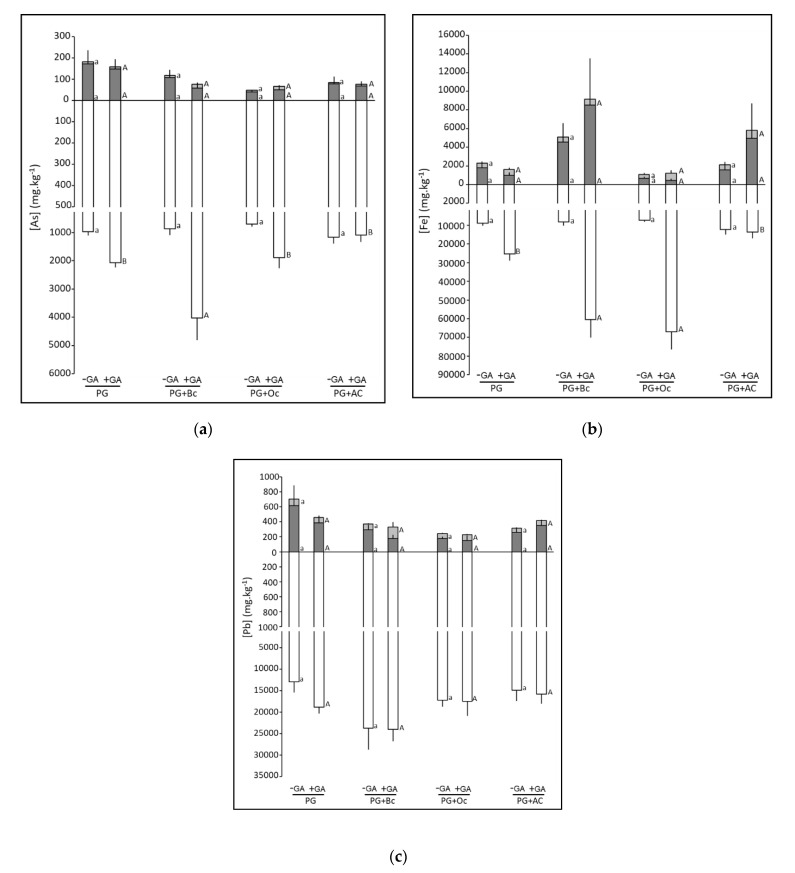
Leaf (light grey), stem (dark grey) and root (white) arsenic (**a**), iron (**b**), and lead (**c**) concentrations (mg.kg^−1^) of *Populus ×. canescens* after 45 days of growth on Pontgibaud technosol alone (PG) or amended with biochar (PG+Bc), ochre (PG+Oc) and activated carbon (PG+AC), associated or not with green amendment (+/− GA). Small letters indicate a significant difference between the treatments without green amendment while capital letters indicate a significant difference between the conditions with the green amendment (*p* < 0.05) (n = 5 ± SE).

Regarding metal(loid) concentrations, leaf and stem As, Fe, and Pb concentrations were not affected, whatever the amendment treatment (Figure 3). Similarly, root Pb concentrations were not affected. However, root As and Fe concentrations were modified by the amendments, but only in +GA conditions, and by the green amendment. More precisely, in −GA conditions, root As and Fe concentrations were not affected by the application of biochar, ochre, and activated carbon to Pontgibaud. In the +GA conditions, root As concentration was doubled by biochar while root Fe concentrations increased following biochar and activated carbon amendment, by 2.6-fold. The green amendment increased root As concentration in PG (2-fold), PG+Bc (4.6-fold) and PG+Oc (2.7-fold) while it increased root Fe concentration in the same conditions, by 2.8-fold, 7.4-fold, and 9.2-fold, respectively (Figure 3, Table S1). 

## 3. Discussion

The addition of biochar, ochre, and activated carbon generally increased SPW pH and EC, and the association with the clover green amendment further increased those parameters. Previous studies demonstrated the positive effects of those amendments on soil pH and EC [23,24,25,26,27,28,29]. Such improvements can be explained by the alkalinity and the elevated EC of the amendments (Table 4) [30,31]. Moreover, functional groups on the carbon-based amendments can interact with and consume protons [31] and basic cations can be released from biochar, activated carbon and red mud surfaces [23,30]. Furthermore, when fresh green amendment is applied to the soil, it decomposes rapidly, which releases diverse salts, especially basic cations, increasing soil EC and pH [30]; although some studies also showed an acidification of the soil with green amendment [32,33]. Finally, soil EC increase can be related to the high ash and mineral contents of the amendments, especially biochar and activated carbon [30,34].

In general, biochar, ochre, and activated carbon amendments decreased As, Fe, and Pb mobility whereas the green amendment increased it. However, amendments had little effect on metal(loid) availability. Such reduction of metal(loid) mobility following the application of biochar, ochre and activated carbon have been previously observed in the studies of Lebrun et al. [29,31] and Wang and Zhou [35]. The first explanation for metal(loid) immobilization is the pH increase induced by the amendments, especially for cations whose mobility decreases when pH increases [31]. In addition, biochar, activated carbon and red mud possess sorption sites on their surfaces for the fixation of metal(loid)s [3,10,28,36]. On the contrary, green amendment addition mobilized metal(loid)s most likely due to the induced increase in dissolved organic matter, which can form soluble complexes with metal(loid)s [35].

Leaf pigments were not affected by biochar, ochre, and activated carbon soil application. Only the addition of the green amendment increased leaf pigments. Usually, plant pigment levels are increased when soil conditions are improved. However, plant pigment levels did not increase, although soil conditions were ameliorated by biochar, ochre and activated carbon. This non-effect can be linked to the iron mobility and availability to plants [37,38]. Indeed, Fe mobility was not affected by biochar, ochre and activated carbon amendments. This was further confirmed by the increase in plant pigments with the green amendment, associated with the rise in Fe mobility. 

Similarly to leaf pigments, plant dry weight was not affected by any of the amendments, not even clover green amendment. These observations were in contradiction with the previously observed positive effect of the tested amendment types on plant growth [33,39,40], due to the amelioration of the soil conditions. However, here, the improvements of the soil conditions, i.e., increase in SPW pH and EC, and reduction of metal(loid) mobility, was not associated with an improvement of poplar growth. This could be related to the low amounts of added amendment, which was not enough to supply nutrients for this particular poplar species. Moreover, although the green amendment supplied nutrients, such as Fe, it was not enough to counterbalance the mobilization of metal(loid) induced.

Finally, plant metal(loid) accumulation was not affected by the addition of biochar, ochre, or activated carbon, although metal(loid)s tended to be immobilized by these amendments. On the contrary, when plants were grown in the presence of green amendment, plant metal(loid) concentrations were higher than without GA. This can be related to the mobilization of metal(loid)s with this amendment, as demonstrated with the SPW data.

## 4. Materials and Methods

### 4.1. Study Site

The soil was sampled from a former silver-lead extraction mine of the district of Pontgibaud (Auvergne-Rhone-Alpes, France). This mine was very active until the 19th century, which lead to an important degradation of the site. After the extraction, important amounts of mine tailings were deposited on the site. These tailings were characterized by a sandy texture, and elevated As and Pb concentrations. Moreover, previous studies showed that soil sampled on this site was acidic (pH 4.6), had a low organic matter content (1.42%), and a low nutrient availability [12,29].

### 4.2. Amendments

Four amendments were used: biochar, an activated carbon, a red mud, and a green amendment.

The biochar was obtained from the slow pyrolysis of hardwood biomass (oak, beech, and charm) at 500 °C with a heating rate of 2.5 °C.min^−1^ and a residence time of three hours. After the pyrolysis, biochar material was sieved to obtain a particle size of 0.5 to 1 mm. The biochar was provided by La Carbonerie (Crissey, France).

The activated carbon was provided by Jacobi Carbons (Paris, France). It was made from a mineral base and activated using steam.

The red mud was an ochre sampled from a charcoal mine in Ales (France). It was dried and crushed into powder prior to use.

These three amendments have been previously characterized [38,41,42]. Characteristics are given in Table 4. 

The last amendment was made of the aerial parts (leaves and stems) of *Trifolium repens* plants grown on the garden soil. Four growing cycles were performed. The aerial biomass was cut 1 cm above soil level, dried, and crushed into powder.

### 4.3. Experimental Design

The three amendments, biochar, activated carbon, and ochre were added either alone or combined with the green amendment, making eight treatments in total: non-amended Pontgibaud (PG, −GA), Pontgibaud amended with 0.4% (*w*/*w*) green amendment (PG, +GA), Pontgibaud amended with 2% (*w*/*w*) biochar (PG+Bc, −GA), Pontgibaud amended with 2% (*w*/*w*) biochar and 0.4% (*w*/*w*) green amendment (PG+Bc, +GA), Pontgibaud amended with 1% (*w*/*w*) ochre (PG+Oc, −GA), Pontgibaud amended with 1% (*w*/*w*) ochre and 0.4% (*w*/*w*) green amendment (PG+Oc, +GA), Pontgibaud amended with 2% (*w*/*w*) activated carbon (PG+AC, −GA), and Pontgibaud amended with 2% (*w*/*w*) activated carbon and 0.4% (*w*/*w*) green amendment (PG+AC, +GA). For each treatment, five pots (11 × 11 × 11 cm) were prepared. After an equilibration period of two weeks, one rooted cutting of *Populus x. canescens* was placed inside each pot (T0). Plants were grown for 45 days in a growing chamber with a 16 h light period and a temperature of 20 °C.

### 4.4. Soil Pore Water Sampling and Analysis

In each pot, one soil moisture sampler (Rhizon®, model MOM, Rhizosphere Research Product, Wageningen, The Netherlands) was placed at 40 °C. At the beginning (T0) and at the end (T45) of the experiment, SPWs were sampled using syringes, after saturation of the pots the day before. On these SPWs, pH, electrical conductivity, and redox potential were measured using a multimeter (Serveur Excellence), after which samples were acidified and As, Fe, and Pb concentrations were measured by Inductively Coupled Plasma–Atomic Emission Spectroscopy (ICP-AES) (ULTIMA2, HORIBA, Labcompare, San Francisco, CA, USA).

### 4.5. Soil Sampling and Analysis

At the end of the experiment—i.e., when plants were harvested—the soil was sampled from each pot, stored in sterile bags, and dried at room temperature. Phytoavailable As, Fe, and Pb concentrations were determined through CaCl_2_ extractions: 1 g of soil was mixed with 10 mL CaCl_2_ (0.01 M) and mixtures were agitated for 2 hours at room temperature (150 rpm, horizontal agitation). Following, solutions were filtered, acidified and As, Fe, and Pb concentrations were measured by ICP-AES.

### 4.6. Plant Harvest and Analysis

After 45 days, pigments were measured on the last leaf having completed its growth using a mobile sensor clip (DUALEX SCIENTIFIC^+TM^, FORCE A, Paris, France). Stem length was also determined. Plants were harvested, roots were thoroughly washed with tap water followed by distilled water in order to remove soil particles adhering to the roots. Leaves, stems, and roots were separated, dried at 60 °C for 72 h and weighted. Finally, plant materials were digested in a microwave in the presence of nitric and hydrochloric acids, and As, Fe, and Pb were measured by ICP-AES.

### 4.7. Statistical Analysis

Data were analyzed using R software version 4.0.2 [43]. For each condition with and without green amendment, the effect of biochar, activated carbon, and ochre was assessed using the following procedure. Normality of data was evaluated using the Shapiro test, then the homogeneity of variance was tested using either the Bartlett test (for normal data) or the Fligner test (for non-normal data). Means were then compared using the ANOVA test for parametric data or the Kruskal–Wallis test for non-parametric data, followed by a posthoc test.

Furthermore, the effect of the green amendment for each treatment was evaluated, using the same procedure, but means were compared through the Student’s *t*-test or the Wilcox test, respectively. Finally, for SPW data, the effect was assessed using the same procedure and paired-tests.

The difference was considered significant at *p* < 0.05.

## 5. Conclusions

This study demonstrated that biochar, ochre, and activated carbon amendments were efficient in reducing soil acidity and metal(loid) mobility. On the contrary, the green amendment reduced soil acidity but mobilized metal(loid)s. However, none of the amendments ameliorated plant growth parameters.

In conclusion, the association of biochar, ochre, or activated carbon with clover green amendment was not efficient for the phytoremediation of As and Pb polluted soils using *Populus x. canescens*.

## Figures and Tables

**Table 1:** Soil pore water physicochemical properties (pH, electrical conductivity (µS.cm^−1^) and redox potential (mV)) determined at the beginning (T0) and at the end of the experiment (T45) in Pontgibaud technosol alone (PG) or amended with biochar (PG+Bc), ochre (PG+Oc) and activated carbon (PG+AC), associated or not with green amendment (+/− GA). Small letters indicate a significant difference between the treatments without green amendment while capital letters indicate a significant difference between the conditions with the green amendment (*p* < 0.05) (n = 5 ± SE).

		pH			Electrical Conductivity (µS.cm^−1^)			Redox Potential (mV)		
		T0	T45	Time Effect	T0	T45	Time effect	T0	T45	Time Effect
PG	−GA	4.7 ± 0.4 b	5.1 ± 0.1 c	Ns	2072 ± 214 b	3961 ± 380 a	**	364 ± 25 ab	387 ± 15 a	ns
	+GA	4.5 ± 0.1 C	5.7 ± 0.3 A	*	2614 ± 107 B	3282 ± 657 A	ns	303 ± 8 A	268 ± 19 AB	ns
PG+Bc	−GA	6.5 ± 0.1 a	6.5 ± 0.0 a	Ns	2754 ± 69 a	3359 ± 433 ab	ns	305 ± 13 bc	293 ± 16 b	ns
	+GA	6.8 ± 0.2 A	6.1 ± 0.2 A	*	3855 ± 198 A	3691 ± 170 A	ns	260 ± 8 B	197 ± 37 AB	ns
PG+Oc	−GA	6.3 ± 0.1 a	6.3 ± 0.1 ab	Ns	3126 ± 171 a	4184 ± 252 a	*	288 ± 19 c	295 ± 10 b	ns
	+GA	6.6 ± 0.1 A	5.6 ± 0.7 A	Ns	3959 ± 226 A	3809 ± 319 A	ns	243 ± 16 B	207 ± 6 B	ns
PG+AC	−GA	4.8 ± 0.2 b	6.2 ± 0.1 b	**	1862 ± 68 b	2322 ± 404 b	ns	380 ± 16 a	297 ± 10 b	**
	+GA	5.7 ± 0.2 B	6.2 ± 0.1 A	*	2330 ± 93 B	2876 ± 223 A	*	247 ± 13 A	270 ± 8 A	ns

Difference between T0 and TF, i.e. time effect, is shown by: * *p* < 0.05, ** *p* < 0.01, ns = non-significant.

**Table 2:** Soil pore water metal(loid) (As, Fe, Pb) concentrations (mg.L^−1^) determined at the beginning (T0) and at the end of the experiment (T45) in Pontgibaud technosol alone (PG) or amended with biochar (PG+Bc), ochre (PG+Oc) and activated carbon (PG+AC), associated or not with green amendment (+/− GA). Small letters indicate a significant difference between the treatments without green amendment while capital letters indicate a significant difference in between the conditions with the green amendment (*p* < 0.05) (n = 5 ± SE).

		[As] (mg.L^−1^)			[Fe] (mg.L^−1^)			[Pb] (mg.L^−1^)		
		T0	T45	Time Effect	T0	T45	Time Effect	T0	T45	Time Effect
PG	-GA	0.19 ± 0.02 a	0.30 ± 0.04 a	ns	0.32 ± 0.04 a	0.27 ± 0.05 a	ns	4.4 ± 0.5 a	3.7 ± 1.0 a	ns
	+GA	0.37 ± 0.05 A	0.58 ± 0.11 A	ns	2.53 ± 0.17 A	28.58 ± 19.69 B	ns	11.8 ± 0.7 A	4.1 ± 0.2 A	***
PG+Bc	-GA	0.07 ± 0.03 b	0.28 ± 0.01 a	ns	0.16 ± 0.00 b	0.26 ± 0.03 a	ns	4.0 ± 0.2 a	2.7 ± 0.2 a	**
	+GA	0.35 ± 0.10 A	1.04 ± 0.41 A	ns	0.34 ± 0.11 B	68.87 ± 34.63 AB	ns	5.9 ± 1.6 BC	0.6 ± 0.4 B	*
PG+Oc	-GA	0.05 ± 0.02 b	0.27 ± 0.00 a	***	0.25 ± 0.06 ab	0.23 ± 0.01 a	ns	3.6 ± 0.3 a	3.7 ± 0.3 a	ns
	+GA	0.05 ± 0.03 B	1.20 ± 0.14 A	**	0.19 ± 0.02 B	143.13 ± 24.49 A	**	6.2 ± 0.3 B	1.2 ± 0.4 B	**
PG+AC	-GA	0.01 ± 0.01 b	0.28 ± 0.01 a	***	0.21 ± 0.02 ab	0.32 ± 0.09 a	ns	4.5 ± 0.3 a	3.3 ± 0.1 a	*
	+GA	0.46 ± 0.05 A	0.93 ± 0.20 A	ns	0.38 ± 0.03 B	17.80 ± 6.60 B	**	4.7 ± 0.3 C	3.6 ± 0.4 A	ns

Difference between T0 and TF, i.e. time effect, is shown by: * *p* < 0.05, ** *p* < 0.01, *** *p* < 0. 001 and ns = non-significant.

**Table 3:** CaCl_2_- extractable metal(loid) (As, Fe, Pb) concentrations (mg.kg^−1^) determined at the end of the experiment in Pontgibaud technosol alone (PG) or amended with biochar (PG+Bc), ochre (PG+Oc), and activated carbon (PG+AC), associated or not with green amendment (+/− GA). Small letters indicate a significant difference between the treatments without green amendment while capital letters indicate a significant difference between the conditions with the green amendment (*p* < 0.05) (n = 5 ± SE).

		[As] (mg.kg^−1^)	[Fe] (mg.kg^−1^)	[Pb] (mg.kg^−1^)
PG	−GA	0.00 ± 0.00 a	3.37 ± 0.70 ab	114 ± 20 ab
	+GA	0.00 ± 0.00 A	3.99 ± 0.61 A	90 ± 6 A
PG+Bc	−GA	0.12 ± 0.08 a	5.06 ± 0.61 a	78 ± 5 b
	+GA	0.04 ± 0.04 A	2.93 ± 0.14 A	109 ± 16 A
PG+Oc	−GA	0.00 ± 0.00 a	2.98 ± 0.24 b	69 ± 5 c
	+GA	0.10 ± 0.09 A	2.67 ± 0.18 AB	77 ± 5 A
PG+AC	−GA	0.00 ± 0.00 a	2.85 ± 0.10 b	124 ± 10 a
	+GA	0.00 ± 0.00 A	2.28 ± 0.03 B	94 ± 6 A

**Table 4:** Amendments (biochar, activated carbon, ochre) properties.

		Biochar	Activated Carbon	Ochre
pH		8.5 ± 0.0	9.5 ± 0.0	8.0 ± 0.1
Electrical conductivity	µS.cm^−1^	302 ± 1	5 ± 0	1595 ± 35
Redox potential	mV	166 ± 9	200 ± 4	261 ± 6
Specific surface area	m^2^.g^−1^	4.38	1050	ND
Total pore volume	cm^3^.g^−1^	0.01	0.87	ND
Mean pore diameter	Nm	9.13	9	ND
Cation exchange capacity	cmol.kg^−1^	0.5 ± 0.2	7.7 ± 0.3	5.9 ± 0.2
C content	%	78.7 ± 1.1	81.9 ± 2.5	1.3 ± 0.3
H content	%	1.7 ± 0.1	0.4 ± 0.0	1.6 ± 0.1
N content	%	2.4 ± 0.8	1.9 ± 0.8	0.6 ± 0.1

ND = not determined.

## Supplementary Materials

The following are available online at https://www.mdpi.com/article/10.3390/plants10071374/s1, Table S1: Green amendment effect on soil pore water (SPW) pH, electrical conductivity (EC), redox potential (Eh) and metal(loid) concentrations determined at the beginning (T0) and at the end (T45) of the experiment; on CaCl_2_-extractable metal(loid) concentrations, plant pigments (nitrogen balance index (NBI), chlorophyll, flavonoid and anthocyanin contents), and plant metal(loid) concentrations. Levels of significance: * *p* < 0.05, ** *p* < 0.01, *** *p* < 0. 001 and ns = non-significant.
