# Supplementary material for: The Potential of Clover Green Amendment, Associated with Biochar, Activated Carbon or Ochre, for the Phytoremediation, Using Populus x. canescens, of a Former Mine Technosol
Source: Plants (Basel). 2021 Jul 5;10(7):1374. doi: 10.3390/plants10071374 (PMC8309311; doi:10.3390/plants10071374)
Supplement: Supplementary file 1 [file plants-10-01374-s001.zip › plants-1279799-supplementary.pptx]

## Slide 1
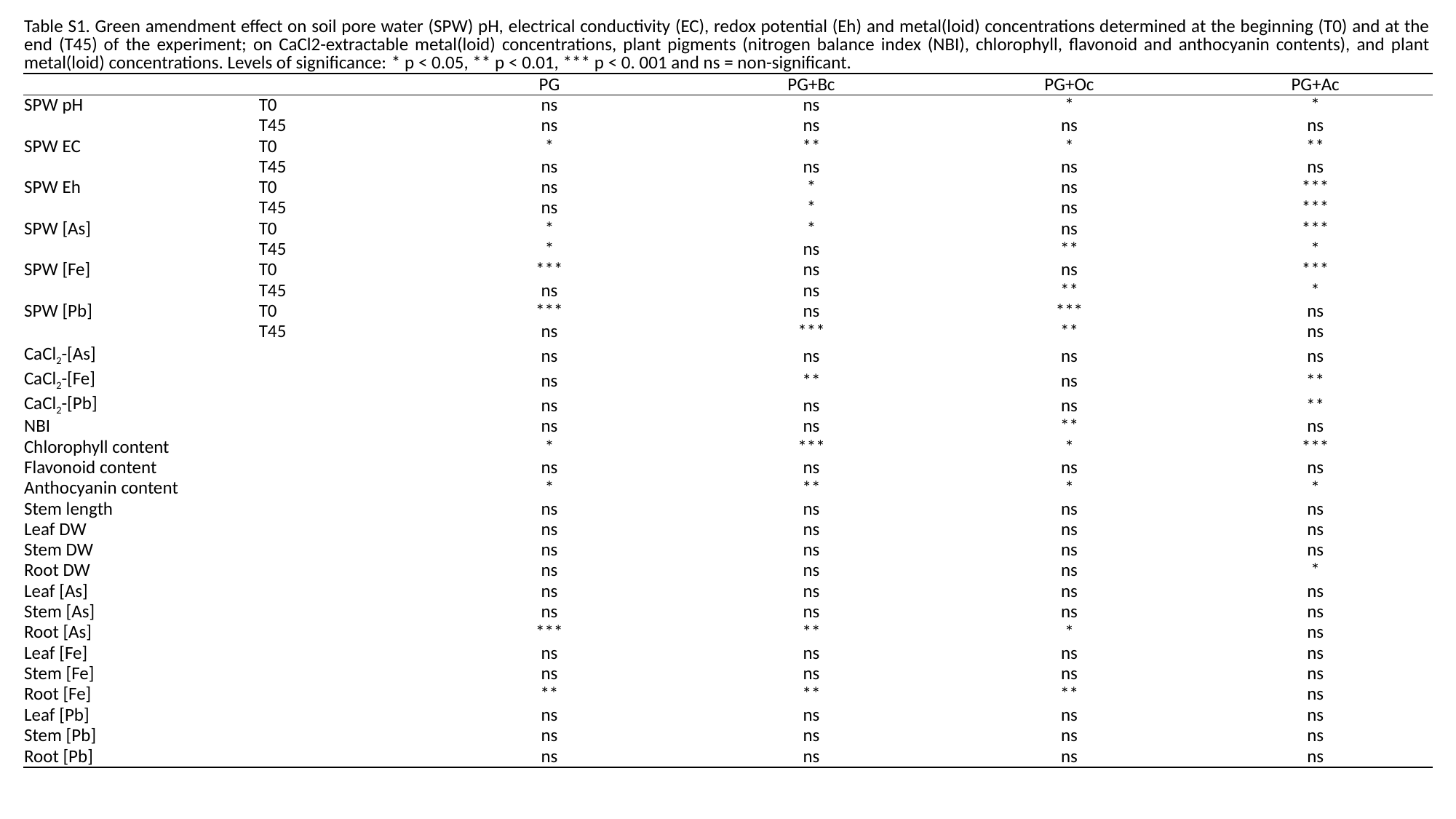

| Table S1. Green amendment effect on soil pore water (SPW) pH, electrical conductivity (EC), redox potential (Eh) and metal(loid) concentrations determined at the beginning (T0) and at the end (T45) of the experiment; on CaCl2-extractable metal(loid) concentrations, plant pigments (nitrogen balance index (NBI), chlorophyll, flavonoid and anthocyanin contents), and plant metal(loid) concentrations. Levels of significance: \* p < 0.05, \*\* p < 0.01, \*\*\* p < 0. 001 and ns = non-significant. | | | | | |
| --- | --- | --- | --- | --- | --- |
| | | PG | PG+Bc | PG+Oc | PG+Ac |
| SPW pH | T0 | ns | ns | \* | \* |
| | T45 | ns | ns | ns | ns |
| SPW EC | T0 | \* | \*\* | \* | \*\* |
| | T45 | ns | ns | ns | ns |
| SPW Eh | T0 | ns | \* | ns | \*\*\* |
| | T45 | ns | \* | ns | \*\*\* |
| SPW [As] | T0 | \* | \* | ns | \*\*\* |
| | T45 | \* | ns | \*\* | \* |
| SPW [Fe] | T0 | \*\*\* | ns | ns | \*\*\* |
| | T45 | ns | ns | \*\* | \* |
| SPW [Pb] | T0 | \*\*\* | ns | \*\*\* | ns |
| | T45 | ns | \*\*\* | \*\* | ns |
| CaCl2-[As] | | ns | ns | ns | ns |
| CaCl2-[Fe] | | ns | \*\* | ns | \*\* |
| CaCl2-[Pb] | | ns | ns | ns | \*\* |
| NBI | | ns | ns | \*\* | ns |
| Chlorophyll content | | \* | \*\*\* | \* | \*\*\* |
| Flavonoid content | | ns | ns | ns | ns |
| Anthocyanin content | | \* | \*\* | \* | \* |
| Stem length | | ns | ns | ns | ns |
| Leaf DW | | ns | ns | ns | ns |
| Stem DW | | ns | ns | ns | ns |
| Root DW | | ns | ns | ns | \* |
| Leaf [As] | | ns | ns | ns | ns |
| Stem [As] | | ns | ns | ns | ns |
| Root [As] | | \*\*\* | \*\* | \* | ns |
| Leaf [Fe] | | ns | ns | ns | ns |
| Stem [Fe] | | ns | ns | ns | ns |
| Root [Fe] | | \*\* | \*\* | \*\* | ns |
| Leaf [Pb] | | ns | ns | ns | ns |
| Stem [Pb] | | ns | ns | ns | ns |
| Root [Pb] | | ns | ns | ns | ns |
| | | | | | |
